# Effectiveness of a Parent-Based eHealth Intervention for Physical Activity, Dietary Behavior, and Sleep Among Preschoolers: Protocol for a Randomized Controlled Trial

**DOI:** 10.2196/58344

**Published:** 2024-09-12

**Authors:** Peng Zhou, Huiqi Song, Patrick W C Lau, Lei Shi, Jingjing Wang

**Affiliations:** 1 Department of Sport, Physical Education and Health Faculty of Arts and Social Sciences Hong Kong Baptist University Hong Kong China (Hong Kong); 2 The Jockey Club School of Public Health and Primary Care The Chinese University of Hong Kong Hong Kong China (Hong Kong); 3 Beijing Normal University-Hong Kong Baptist University United International College Zhuhai China; 4 Mass Sports Research Center China Institute of Sport Science Beijing China

**Keywords:** physical activity, dietary behavior, sleep, electronic health, eHealth, preschoolers, parenting

## Abstract

**Background:**

Preschoolers’ lifestyles have become physically inactive and sedentary, their eating habits have become unhealthy, and their sleep routines have become increasingly disturbed. Parent-based interventions have shown promise to improve physical activity (PA), improve dietary behavior (DB), and reduce sleep problems among preschoolers. However, because of the recognized obstacles of face-to-face approaches (eg, travel costs and time commitment), easy access and lower costs make eHealth interventions appealing. Previous studies that examined the effectiveness of parent-based eHealth for preschoolers’ PA, DB, and sleep have either emphasized 1 variable or failed to balance PA, DB, and sleep modules and consider the intervention sequence during the intervention period. There is an acknowledged gap in parent-based eHealth interventions that target preschoolers raised in Chinese cultural contexts.

**Objective:**

This study aims to investigate the effectiveness of a parent-based eHealth intervention for PA, DB, and sleep problems among Chinese preschoolers.

**Methods:**

This 2-arm, parallel, randomized controlled trial comprises a 12-week intervention with a 12-week follow-up. A total of 206 parent-child dyads will be randomized to either an eHealth intervention group or a control group. Participants allocated to the eHealth intervention group will receive 12 interactive modules on PA, DB, and sleep, with each module delivered on a weekly basis to reduce the sequence effect on variable outcomes. The intervention is grounded in social cognitive theory. It will be delivered through social media, where parents can obtain valid and updated educational information, have a social rapport, and interact with other group members and facilitators. Participants in the control group will receive weekly brochures on PA, DB, and sleep recommendations from kindergarten teachers, but they will not receive any interactive components. Data will be collected at baseline, 3 months, and 6 months. The primary outcome will be preschoolers’ PA. The secondary outcomes will be preschoolers’ DB, preschoolers’ sleep duration, preschoolers’ sleep problems, parents’ PA, parenting style, and parental feeding style.

**Results:**

Parent-child dyads were recruited in September 2023. Baseline and posttest data collection occurred from October 2023 to March 2024. The follow-up data will be obtained in June 2024. The results of the study are expected to be published in 2025.

**Conclusions:**

The parent-based eHealth intervention has the potential to overcome the barriers of face-to-face interventions and will offer a novel approach for promoting a healthy lifestyle among preschoolers. If this intervention is found to be efficacious, the prevalence of unhealthy lifestyles among preschoolers may be alleviated at a low cost, which not only has a positive influence on the health of individuals and the well-being of the family but also reduces the financial pressure on society to treat diseases caused by poor lifestyle habits.

**Trial Registration:**

ClinicalTrials.gov NCT06025019; https://clinicaltrials.gov/study/NCT06025019

**International Registered Report Identifier (IRRID):**

DERR1-10.2196/58344

## Introduction

### Background

People’s lifestyles affect their health and well-being [1]. The main lifestyle dimensions of physical activity (PA), dietary behavior (DB), and sleep have played pivotal roles in decreasing cardiovascular risk [2], improving brain growth velocity [3], preventing mental disorders [4], improving social interactions [5], and improving the central nervous system [6]. These dimensions appear to be interrelated. PA brings about substantial benefits in not only reducing high-fat calorie consumption [1] but also improving sleep patterns and increasing sleep duration [7]. A longer nighttime sleep duration was shown to be associated with a less frequent intake of fast foods and sugar-sweetened beverages [8], and people with sufficient sleep quality were more likely to form active lifestyles [9]. In the past few decades, individuals’ lifestyles have become physically inactive and sedentary [10], their eating habits have become unhealthy [11], and their sleep routines have become increasingly disturbed [12]. These changes have led to an ever-increasing prevalence of noncommunicable diseases such as diabetes and obesity [13]. Therefore, an effective intervention is needed to reverse this negative trend. A previous study indicated that children of preschool age may respond better to treatment as the lifestyle behaviors in this period are malleable and less entrenched [14]. Lifestyles formed in the early years of life can extend throughout the lifetime and influence lifelong health trajectories [15].

Parents have a profound impact on the healthy lifestyle of preschoolers and are the key agents of behavioral modification [16]. Parents provide an overwhelming majority of cues for PA, diet, and sleep routines and are responsible for role modeling these behaviors among their children [17]. Preschoolers are particularly dependent on their parents since younger children have limited autonomy and rely on parental supervision [18]. A longitudinal study demonstrated that parents’ influence on children’s lifestyle may be attenuated as children age because peer interaction gradually becomes their preferred activity when they grow up [19].

Despite the importance of parental involvement, previous parent-based interventions involving home visits and in-person education have been associated with high attrition rates, with many relying on self-reported assessments, failing to use a randomized controlled study design [20], and showing lower recruitment [21]. Other limitations of delivering interventions face-to-face within the home are related to the associated time commitment for participants [21], the need for childcare for other siblings [22], stigma, parental denial [23], the need to travel [24], and associated costs [23], which have hindered intervention adoption and adherence and have ultimately decreased the effectiveness of these programs. In response, eHealth, which is defined as the cost-effective and secure use of information and communication technologies in support of health, including health care services, health surveillance, health literature, health education, health knowledge, and health research [25], has the potential to address these barriers [26], providing convenience and flexibility for parents and enabling parents to participate regardless of their geographic location [27]. Moreover, personalized information and feedback delivered via eHealth interventions may augment behavioral change strategies [28].

Efficacy trials assessing the effectiveness of parent-based eHealth for preschoolers’ PA, diet, and sleep have been conducted in 12 studies [29-40]. Although these 12 studies demonstrated that participants engaging in eHealth interventions had significant improvements in their healthy lifestyles, 7 studies [29,33,35-38,40] only concentrated on 1 variable, with 2 studies focusing on sleep, 3 focusing on fruit and vegetable intake, and 2 focusing on PA. A study by Ling et al [34] reported that disclosed intervention content between 2 groups may result in insignificant group-by-time changes in PA and diet. The remaining 4 studies [30-32,39] found a significant group-by-time difference in diet, but this effect was not found in PA and sleep. Possible reasons for this may be that these 4 studies involved more diet modules compared to PA and sleep modules. Overall, previous studies that examined the effectiveness of parent-based eHealth on preschoolers’ PA, diet, and sleep have either emphasized 1 variable or failed to balance PA, diet, and sleep modules and consider the intervention sequence during the intervention period. Specifically, more exposure to diet modules relative to fewer PA modules may yield outcomes favoring diet variables. When the posttest was conducted, parent-child dyads may have been more familiar with the modules distributed at the end of the intervention period, thus producing favorable outcomes regarding the content of these modules compared to others.

Empirical relationships obtained in one culture may be a product of its milieu and may not be generalizable to other countries [41]. Very little is currently known about whether parent-based eHealth can effectively improve preschoolers’ PA and diet and reduce sleep problems in China. In the past decade, the prevalence of physical inactivity, unhealthy diet patterns, and sleep problems among Chinese children has increased. Around 70.1% of preschoolers do not meet the PA guidelines recommended by the World Health Organization (WHO) (ie, over 180 minutes of PA daily, including at least 60 minutes of moderate-to-vigorous PA) [42]. The total energy consumption involving fat and sodium exceeds the Chinese Recommended Nutrient Intakes by 10% and 56%, respectively [43]. Over 61% of Chinese preschoolers experienced or are experiencing insufficient sleep and sleep problems (eg, sleep onset latency) [44]. China has the largest population in the world, and the implementation of a traditional face-to-face intervention to improve the unhealthy lifestyles of preschoolers would pose a heavy financial burden on society [44]. An effective and feasible intervention strategy is imperative to tackle these growing health problems.

Currently, the number of smartphone and internet users in China is 1.64 billion and 1028.74 million, respectively [45]. eHealth interventions addressing unhealthy lifestyles could be cost-effective and easily scaled up [46]. Furthermore, affected by Confucianism, Chinese parents emphasize “training” (Guan), which refers to parents’ control and demands [47]. “Guan,” a typical characteristic of Chinese parenting, may generate a profound impact on determining a child’s lifestyle behaviors, including PA, diet, and sleep [48]. Considering the shortcomings revealed in the study design of previous eHealth studies, high-quality robustly designed research studies that balance the intervention content and sequence are needed to determine the effectiveness of eHealth interventions to support behavior change in Chinese preschoolers, with parents being agents of change.

### Research Objective

This study aims to investigate the effectiveness of parent-based eHealth interventions for PA, DB, and sleep among Chinese preschoolers.

## Methods

### Study Design

**Figure 1:**
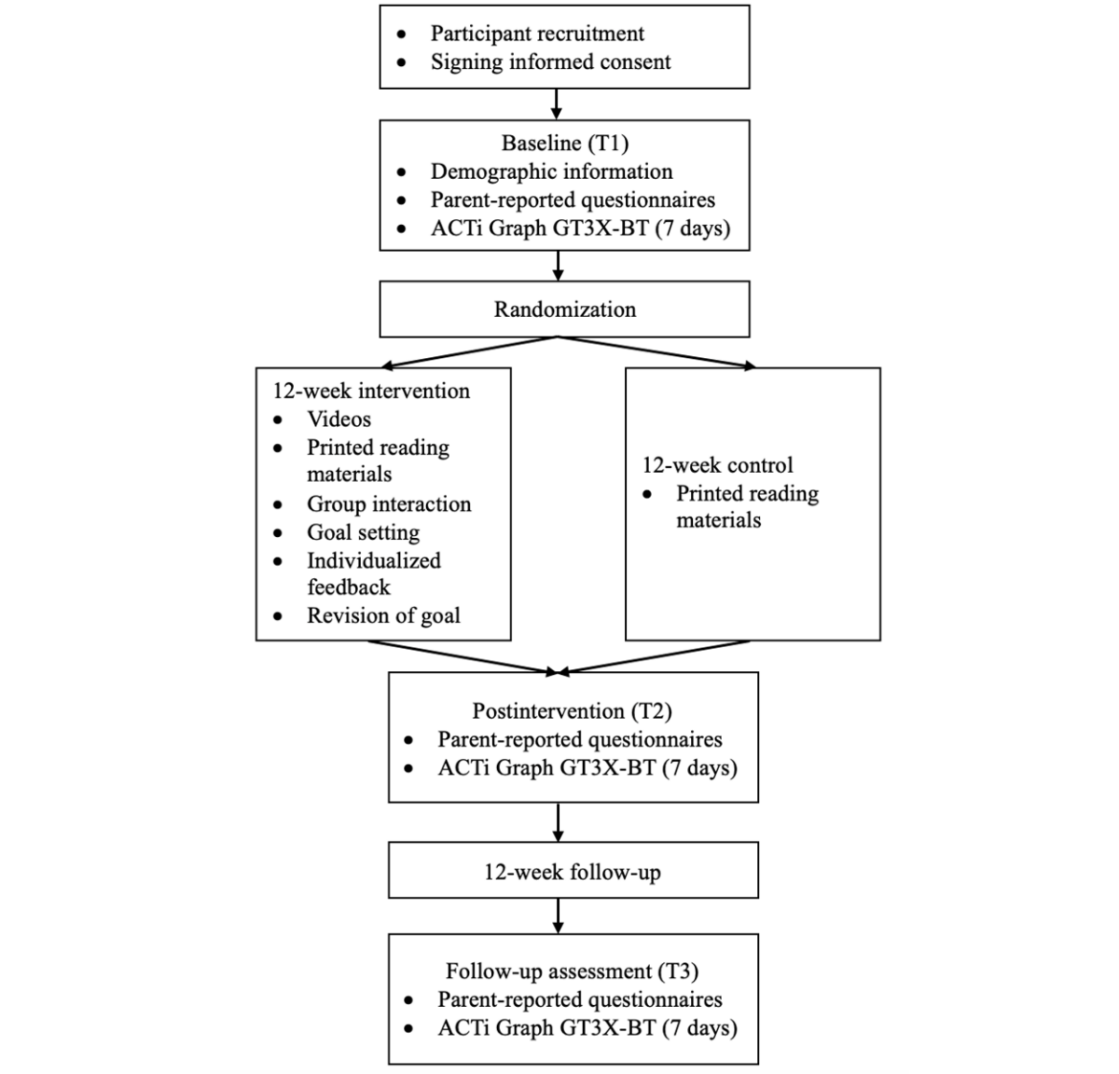
Study design.

This study is a 2-arm, parallel, single-blinded, randomized controlled trial designed to promote preschoolers’ PA, DB, and sleep by providing parents with relevant health information through an eHealth modality. The first arm involves an eHealth intervention, which will deliver evidence-based information and interactive components via social media (ie, WeChat, similar to WhatsApp; and TikTok or Douyin, similar to YouTube). The interactive components include push notifications, social rapport, influence agents, goal setting, and personalized feedback. The second arm involves a control intervention, which consists of printed evidence-based educational materials on standard recommendations for PA, diet, and sleep, without interactive components. The primary outcome will be preschoolers’ PA. The secondary outcomes will be preschoolers’ DB, preschoolers’ sleep duration and sleep problems, parents’ PA, parenting style, and parental feeding style. The intervention will last 12 weeks, with a 12-week follow-up. Based on the results of a previous systematic review, the duration of parent-based eHealth interventions varied from 8 weeks to 2 years, with over half of the studies being shorter than 12 weeks [49]. In this case, the 12-week intervention duration, which roughly coincides with the length of a school semester in the Chinese educational system of kindergarten (ie, two 3-month semesters in an academic year), is considered sufficient for behavioral modification. The proposed study design is shown in Figure 1. The study will follow the CONSORT-EHEALTH (Consolidated Standards of Reporting Trials Statement for Randomized Trials: Improving and Standardizing Evaluation Reports of Web-Based and Mobile Health Interventions) guidelines [50].

### Ethical Considerations

The study has been approved by the Hong Kong Baptist University Research Ethics Committee (reference number: SOSC-SPEH-2022-23_115). This study has been prospectively registered at the ClinicalTrials.gov Protocol Registration and Results System (PRS) (NCT06025019). Written informed consent will be obtained from all participants or their legal guardians. Personal data (ie, gender, age, and marital status); preschoolers’ PA, diet, and sleep behaviors; and family information (family economic status, parents’ education level, and family structure) will be collected. No other information related to participant identity will be recorded or disclosed. The data collected by the researchers will be destroyed after the project is completed and published (within 7 years). In case of withdrawal, the data will be deleted from the data file immediately. Incentives (honorary certificates) will be offered to families judged to be more active and healthier.

### Participant Recruitment and Eligibility Criteria

Parent-child dyads will be recruited from kindergartens in Guiyang, Guizhou Province, China, with the support of Sun Yat-sen University and Guiyang Preschooler Education College. The entire recruitment process will be school-based. Kindergarten teachers will be asked to send a message to the parents in their respective WeChat class group. The message will briefly introduce the objectives and significance of the study. Parents will respond “yes” or “no” regarding their intention or willingness to participate. Face-to-face meetings will be held to provide more details to those parents who agree to participate in the study, especially on measurements, and to clarify the benefits that parents will receive after the study. Parents will be asked to add the researcher’s WeChat and sign the informed consent at the end of parent meetings. The inclusion criteria are as follows: (1) parents are older than 21 years and have children aged 3-6 years (the age range from 3 to 6 years has been found to be a strong predictor of future health) [51], (2) parental commitment to participate in the overall 6-month intervention, (3) parents have access to mobile technology, (4) parents and children are healthy (referring to a state of physical, mental, social, intellectual, and emotional well-being and the absence of disease) based on a health assessment performed by the kindergarten on the commencement day of a new semester, and (5) parents reside with participating children for at least 4 days a week in order to adequately expose the children to the parent-based intervention.

### Randomization

Once participants are recruited and baseline measurements are collected, participants will be randomly allocated to either an intervention group (eHealth group) or a control group (traditional group) in a 1:1 ratio through a computer-generated list, using the statistical software SPSS (IBM SPSS 27, IBM Corp). The randomization will be conducted by a researcher who is blinded to the intervention recruitment and delivery. Random allocation to either the eHealth group or the control group will not be informed to parents.

### Theoretical Foundation

**Figure 2:**
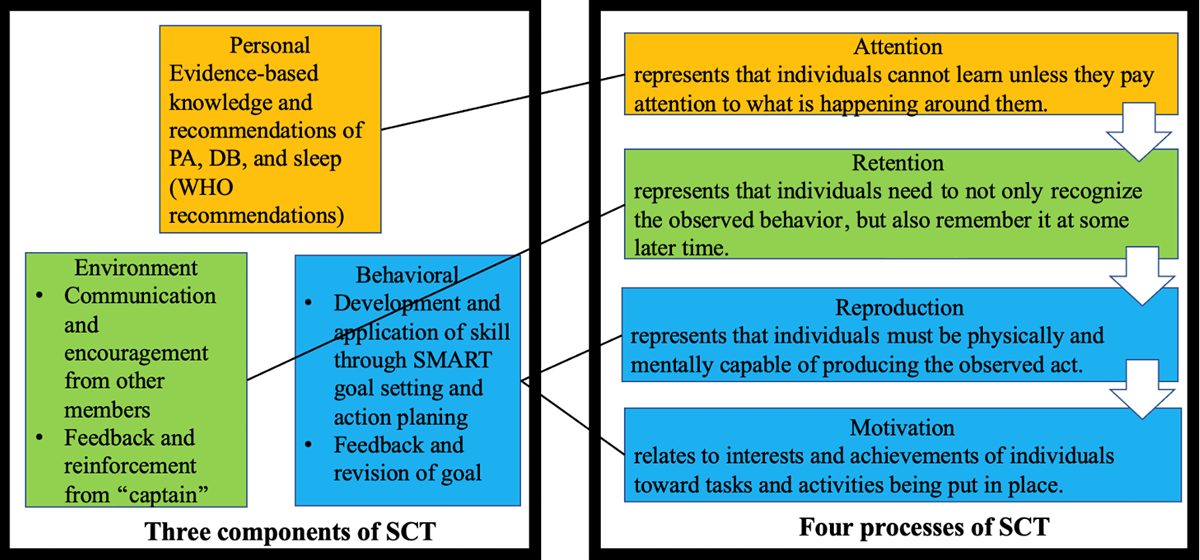
SCT-based eHealth intervention. DB: dietary behavior; PA: physical activity; SCT: social cognitive theory; SMART: Specific, Measurable, Achievable, Relevant, and Time-bound; WHO: World Health Organization.

The intervention is grounded upon social cognitive theory (SCT), which considers learning as a social phenomenon that comes from a person’s interaction with others through watching and observing [52]. Moreover, it considers the evidence that eHealth implemented directly to preschoolers may have adverse impacts on preschoolers’ healthy lifestyles (eg, disturbed sleep, myopia, and increased screen time). Thus, a key component of this intervention is to involve parents in modeling health-promoting behaviors among their preschool-aged children. Figure 2 depicts how the intervention will be conducted. First, SCT proposes that an individual’s behavioral modification can be influenced by personal, environmental, and behavioral factors [53]. These 3 factors describe what intervention content should be involved to induce behavioral modification:

- Personal factors refer to an individual’s self-efficacy to carry out a behavior, which is dependent on personality, knowledge, beliefs, self-perceptions, and expectations. Knowledge and beliefs of the importance of PA, DB, and sleep for preschoolers will be addressed through the topic content of the eHealth intervention (videos and reading materials).
- Environmental factors refer to supportive environments that help an individual to conduct a behavior. Interaction with others by posting photos, videos, and ideas concerning a healthy lifestyle will provide participants with vicarious learning. Communication, feedback, and reinforcement from other parents through social media platforms and from research staff through personalized interaction can provide parents with opportunities to practice the skills, potentially contributing to behavioral modification.
- Behavioral factors refer to the responses by the individual once they have practiced carrying out a behavior. The parent will put the skills and behaviors into practice after goal setting and action plans. Positive reinforcement will be gained by monitoring progress with the goals and action plans and the personal benefits experienced.

Additionally, the 4 processes for behavior change involved in SCT are attention, retention, reproduction, and motivation. If any one of these steps is missing, the learning and adaptation of new behaviors will not take place. These 4 steps describe what intervention sequence should be followed to induce behavioral modification:

- Attention, which is matched with personal factors, represents that individuals cannot learn unless they pay attention to what is happening around them. Therefore, attention is tackled by providing printed educational materials and TikTok videos delivered through WeChat [54].
- Retention, which is matched with environmental factors, represents that individuals must not only recognize the observed behaviors but also remember these behaviors at some later time. Therefore, retention is addressed by interactions with other participants through the WeChat group to support parents in remembering the content of videos and educational material. Moreover, each week, parents will receive 4 push notifications to remind them about the information of each interactive component (ie, video or printed material, communication, goal setting, and feedback).
- Reproduction, which is matched with behavioral factors, represents that individuals must be physically and mentally capable of producing the observed behaviors. Therefore, reproduction will be achieved through goal setting based on the SMART (Specific, Measurable, Achievable, Relevant, and Time-bound) goal framework, action plans, and solutions to barriers.
- Motivation, which is matched with behavioral factors, relates to the interests and achievements of individuals regarding the tasks and activities being put in place. Therefore, motivation is addressed by creating cognitive dissonance with parents describing current behaviors and asking parents to identify the positive consequences and expectations as a result of performing the planned behaviors.

### Intervention Procedure

**Figure 3:**
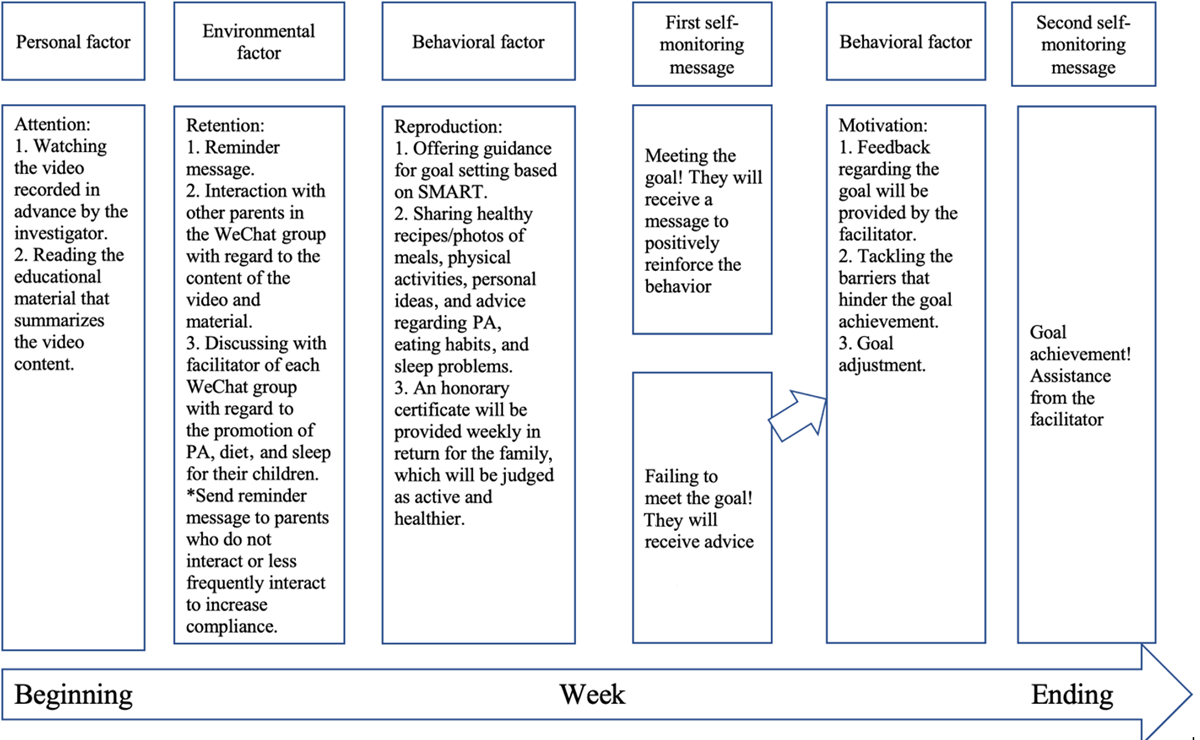
Intervention mapping process of each module based on the 4 steps of social cognitive theory. PA: physical activity; SMART: Specific, Measurable, Achievable, Relevant, and Time-bound.

The eHealth intervention comprises 12 interaction modules, including PA (n=4), DB (n=4), and sleep (n=4). Each module will be rotated weekly to reduce the order effect on the outcome variables during the 12-week intervention period (ie, PA, diet, sleep, PA, diet…). Given that previous social media studies have indicated that 5 to 9 members in a group will produce the desirable group interactive effect [55], the participants will be grouped into 26 WeChat groups (8 members involved in each WeChat group) by the researchers according to the sample size calculation. Given that a facilitator has been found to be important to potentially make the intervention more individualized and may be successful in behavioral change [56], a teacher with expertise in PA, DB, and sleep who is working in the kindergarten will act as a facilitator, also known as a “captain,” in each WeChat group. Facilitators will be trained in a 5-hour workshop before the intervention and will be blinded to the study objectives. They will be responsible for sending videos; facilitating group interactions; responding to questions from parents; helping parents establish PA, diet, and sleep goals for their children; and providing feedback. Parents in each WeChat group will receive 2 self-monitoring text messages, and they will be required to provide a “yes” or “no” response. The first message will be sent as a feedback message to evaluate the progress toward the child’s weekly goal in the middle of the week. An “N” response may elicit a skill-building response to adjust the goal or adopt alternatives to achieve the goal (eg, try offering the same vegetable many times in different ways). If the parent responds “Y,” the facilitator will generate a message to reinforce positive behaviors, such as “Be proud of yourself, you’re getting your child off to a healthy start!” The second self-monitoring message will be sent on the weekend to assess goal attainment for the weekly target behavior before the new weekly theme is introduced. Figure 3 illustrates the intervention process of each module [57,58]. Multimedia Appendix 1 indicates what specific content will be shared.

Consistent with SCT [52], the content of each module will follow 4 steps (Figure 2 illustrates the intervention process of each module) as follows:

1. Attention (read educational material and watch a TikTok or Douyin 3-minute video): Each video involves behavior change techniques, which are mapped onto SCT and are critical for promoting behavior change [59-61]. It will be sent to parents at the beginning of each week, with each video containing information related to goal setting, action plan, feedback, monitoring, shaping knowledge, natural consequences, repetition and substitution, comparison of outcomes, reward and threat, antecedents, identity, and self-belief [54,59].

2. Retention (parent interaction): A meta-analysis assessing the psychological architecture and adherence factors of online interventions indicated that intervention adherence is more likely to be influenced by push factors, peer communications, and personal contact [62]. Parents will receive regular messages to remind them to watch videos and read text. Facilitators and researchers will regularly share questions and images related to the week’s topic, aiming to stimulate discussion among parents [30]. For example, “What is your child’s current PA pattern?” and “Do you read the ingredient label of food when buying?” It was anticipated that parents would gain the most benefit from actively participating (posting information or commenting) based on findings from previous studies on online health discussion groups, which have indicated that parents who wrote posts, commented, asked questions, and provided support to others achieved greater benefits than passive parents [63,64]. If there are parents in the WeChat group who do not post or share, they will receive a reminder message sent by the facilitator via WeChat privately.

3. Reproduction (set goals and conduct behaviors): The facilitator of each WeChat group will help parents set goals related to the weekly topic based on SMART principles [65]. Goals should be individualized and can be adjusted along with the child’s and parent’s progress in behavior changes. The facilitator of each WeChat group will inform the parents via private text messages on how to employ goal setting to help their children set PA, DB, and sleep goals. It is encouraged to share goal achievements with other members in the WeChat group or with the facilitator privately. Throughout the intervention period, parents will be offered the possibility to interact with the facilitator on a regular basis by means of WeChat in order to discuss problems and raise questions regarding the promotion of PA, a healthy diet, and sleep for their children.

4. Motivation (feedback and making changes): Feedback and subsequent revision of goals have been shown to be positively related to successful interventions of behavioral modification [66,67]. Tailored feedback will be provided by a research assistant with expertise in lifestyle (PA, DB, and sleep) based on the goal, barrier identification, and solution. The research assistant will assist parents to revise their weekly theme-related goal following the SMART framework [68].

### Control Group

Parent-child dyads assigned to this group will receive a weekly pamphlet on PA, DB, and sleep recommendations via the child, but they will not receive interactive components.

### Sample Size

A recent systematic review and meta-analysis indicated a small but significant effect size for interventions to improve the PA of children aged younger than 5 years [69]. Interventions that aimed to enhance DB and reduce sleep problems among preschoolers had small to medium effect sizes [9,70]. Based on the information, a priori analysis will be conducted using G*power 3.1.9.6 [71]. A minimum sample size of 82 parent-child dyads in the intervention and control groups separately is required to detect a small effect size (*d*) of 0.3 with a power of 0.80 under the condition of an α level of .05. Considering a 20% potential attrition rate, 206 parent-child dyads in total are needed.

### Outcomes

Data collection will be conducted at baseline (1 week before the intervention; T1), 12 weeks postintervention (at the end of the intervention; T2), and at the end of the 12-week follow-up period (T3).

### Demographic Information

The following demographic information will be collected from parents: socioeconomic status (range of income, educational level, and occupational status), age, gender, and marital status.

The following demographic information will be collected from preschoolers: sex, age, weight, height, and BMI. Preschoolers’ weight will be measured with standard practices to the nearest 0.1 kg using calibrated measurement scales (Salter) and height will be measured using a stadiometer (SECA) to the nearest 0.1 cm. BMI will be calculated with height and weight measurements. As sibship size has been observed to influence parenting behaviors, the number of children in the household will be collected.

### Primary Outcome

#### Preschoolers’ PA

The PA level and sedentary time will be objectively monitored using a triaxial accelerometer ActiGraph GT3X-BT (ActiGraph). The ActiGraph accelerometer has been found to be valid and reliable to objectively measure PA levels in preschoolers [72]. The kindergarten teachers and parents will receive written and video instructions for the use of the accelerometer. In addition, parents will be asked to register an activity diary for both wear and nonwear times. The accelerometer wear will be checked by teachers on each school day. An accelerometer will be affixed to the participant’s right wrist to monitor all activities for a period of 7 continuous days, except during periods of water-related activities or situations involving the risk of damage to the device, such as contact sports or self-defense. Data will be downloaded and initialized using the ActiLife software (version 6.13) and analyzed in 1-second epochs. Valid wear time will be considered at least 16 hours of wear time over at least 3 days (2 weekdays and 1 weekend day) [73]. Nonwear time will be defined by 60 consecutive minutes of 0 counts per minute (CPM), using ActiLife standard approaches. The accelerometer’s activity counts will be categorized into different intensities (ie, sedentary behavior, light PA, moderate PA, and vigorous PA) by using cutoff points as follows: sedentary, <819 CPM; light, 820-3907 CPM; moderate, 3908-6111 CPM; and vigorous, ≥6112 CPM.

### Secondary Outcomes

#### Preschoolers’ DB

Preschoolers’ DB will be assessed using the Children’s Eating Behavior Questionnaire (CEBQ), which has been shown to be valid in Chinese preschool-age children [74]. Parents will rate the frequency of their children’s eating behaviors on a 5-point scale (1=never, 2=rarely, 3=sometimes, 4=often, 5=always) in terms of 8 domains (35 items): satiety responsiveness (eg, My child gets full before the meal is finished), slowness in eating (eg, My child finishes the meal quickly), food fussiness (eg, My child enjoys tasting new foods), food responsiveness (eg, If allowed, my child would eat too much), enjoyment of food (eg, My child enjoys eating), desire to drink (eg, If given the chance, my child would always be having a drink), emotional undereating (eg, My child eats more when happy), and emotional overeating (eg, My child eats more when there is nothing else to do). The scale has high internal consistency and reliability (overall Cronbach α >.7).

#### Preschoolers’ Sleep Duration and Problems

The ActiGraph accelerometer will be used to examine children’s sleep duration in conjunction with a parent-reported log sheet and nap schedule provided by kindergartens. Bedtime and wakeup time will be identified based on the algorithm by Sadeh et al [75], and periods of sleep will be estimated using the algorithm by Tudor-Locke et al [76]. The nighttime sleep and daytime nap durations, bedtime, and wakeup time will be assessed using ActiLife software (version 6.13) in 60-second epochs and matched with the nap schedule filled out by kindergarten teachers and log sheets finished by parents. The total duration of sleep per day will be the sum of nighttime and daytime sleep.

The Chinese version of the Children’s Sleep Habits Questionnaire (CSHQ) [77], a parent survey regularly used to track kids between the ages of 4 and 10 years, will be used to assess preschoolers’ sleep problems. It contains 33 items from 8 domains: bedtime resistance, sleep onset delay, sleep duration, sleep anxiety, night waking, parasomnia, sleep-disordered breathing, and daytime sleepiness. Each scored question is rated on a 3-point scale as follows: 3=“usually if something occurs 5 or more times in a week,” 2=“sometimes if something occurs 2-4 times in a week,” and 1=“rarely if something occurs never or 1 time during a week.” Higher scores represent greater sleep problems. This survey has shown good reliability (Cronbach α=.73) and validity.

#### Preschoolers’ Screen Time

Parents will be asked to answer questions that estimate the usual amount of screen time for their children on a typical weekday and weekend to determine the average screen time per week. Questions also involve the availability of screens and rules about screen entertainment. This questionnaire has been used in a previous study [23].

#### Parents’ PA

Parents’ PA will be assessed using the Chinese version of the International Physical Activity Questionnaire-Short Form (IPAQ-SF). The questionnaire will ask about the time spent being physically active in the last 7 days through 7 questions. The obtained data will be converted to metabolic equivalent task (MET) scores for each dimension or intensity of PA. To calculate the weekly PA (MET-min/week), the number of total minutes dedicated to each activity class is multiplied by the specific MET score for the activity, with 3.3 METs for walking, 4 METs for moderate PA, 6 METs for cycling, and 8 METs for vigorous PA. This questionnaire has been shown to be a reliable and validated measure for assessing PA levels in Chinese cities (overall Cronbach α=.788) [78].

#### Parenting Style

The Parenting Style & Dimension Questionnaire, a self-report instrument designed to measure authoritarian (eg, I yell when I disapprove of my child’s behavior), authoritative (eg, I am responsive to my child’s feelings and needs), and permissive (eg, I find it difficult to discipline my child) parenting styles of the parents of children aged 4-12 years, will be used to evaluate the parenting styles. This scale comprises 32 items, with each item of the scale evaluated using a 5-point Likert scale (“never,” “once in a while,” “about half of the time,” “very often,” and “always”). The overall Cronbach α is .87 [79].

#### Parental Feeding Style

The parental feeding style will be assessed using the Chinese version of the Parent Feeding Style Questionnaire, which consists of 4 parts: instrumental feeding (4 items), emotional feeding (5 items), prompting or encouragement to eat (8 items), and control over eating (10 items). Respondents will be asked to choose responses on a 5-point Likert scale (ranging from “never” to “always”). For example, “I allow my child to choose which food to have for meals, I encourage my child to look forward to the meal.” The average score on each scale will be calculated, and a higher score will be considered to indicate a greater tendency for parents to feed their children in that style [80]. The overall Cronbach α is .75.

#### Parents’ Self-efficacy

Parents’ self-efficacy will be assessed using a previously used questionnaire [23], which has been translated back-to-back into simplified Chinese language. This questionnaire consists of 11 questions related to a child’s PA (4 items), DB (6 items), and sleep (1 item). For example, “How confident are you that you can promote healthy eating habits for your child?” Respondents will be asked to select from a 10-point Likert scale (ranging from “not at all” to “to a very high degree”). A higher score for the sum of the 11 questions will be considered to indicate a higher self-efficacy of parents regarding their children’s PA, DB, and sleep.

### Statistical Analysis

All statistical analyses will be conducted using IBM SPSS 27 (IBM Corp), and a 2-sided *P* value <.05 will be considered statistically significant. Baseline characteristics between the intervention group and control group will be compared using the chi-square test for categorical variables and ANOVA for continuous variables. A mixed model, which accounts for missing repeated measures, will be adopted with a 2-level between-group factor (intervention and control) and 2-level within-group factor (postintervention and follow-up assessment) repeated measures analysis of covariance (RM ANCOVA) to examine whether the eHealth-based parenting intervention improves preschoolers’ PA, DB, and sleep compared to the findings in the control group.

## Results

Parent-child dyads were recruited in September 2023. Baseline and posttest data collection occurred from October 2023 to March 2024. The follow-up data will be obtained in June 2024. The research team has completed data collection for 238 parent-child dyads and is currently analyzing the outcomes. The results of the study are expected to be published in 2025.

## Discussion

Low compliance with a healthy lifestyle (eg, PA, healthy dietary patterns, and sufficient sleep) exacerbates the likelihood of noncommunicable diseases such as obesity [81]. Focusing preventive efforts on early childhood growth trajectories provides an opportunity to address unhealthy lifestyles before individuals’ lifestyles are entrenched [82]. Chinese parents have high standards and a tendency to control their children, and these strong parenting influences may affect the early life behaviors of Chinese children [83]. Given the current economic climate, time constraints are likely to intensify for parents, making face-to-face interventions more problematic. The intervention in this study will be delivered in an easy-access setting (ie, WeChat; monthly active users have reached 1.26 billion, accounting for 77% of internet users aged from 16 to 64 years). Moreover, 70.2% of Chinese people rank Douyin, known as TikTok in Western countries, as the second favorite social media platform [84] on which parents are already engaged and are likely to share what they learn through peer communication. Unfortunately, proven parent-based eHealth interventions against preschoolers’ unhealthy lifestyles are not being practiced routinely in China. Therefore, the objective of this study is to assess the effectiveness of an eHealth intervention for promoting Chinese preschoolers’ PA, DB, and sleep, where parents are the agents of change. This study draws on the success of previous studies. A randomized controlled trial remains the gold standard for evidence of the effectiveness of eHealth interventions [85]. Previous studies have indicated that a longer intervention is more effective than a shorter intervention, possibly because a longer intervention duration allows program content to be repeated and allows participants to internalize program materials [10,86]. According to a previous systematic review, the intervention duration varied from 8 weeks to 2 years, with over half of the studies being shorter than 12 weeks [49]. In China, preschoolers attend kindergarten for 2 semesters over 1 year, with each semester lasting around 12 weeks. Gardner et al [87] indicated that behavioral modification needs at least 2 to 3 months. As such, the 12-week intervention duration in this study, which fits well in the semester time, may be acceptable and feasible for parents and preschoolers. The waitlist control group is identified as having “no intervention,” which is likely to be unacceptable to participants and can contribute to poor retention and follow-up, thus compromising the study’s integrity [88]. A follow-up test can help assess whether any behavioral changes resulting from the parent-based eHealth intervention are sustained over time [89]. Considering that different educational levels (ie, lower literacy vs better literacy) are associated with a poorer understanding of the health information provided, the use of video in this study may be beneficial for parents with lower educational backgrounds [90]. Some new delivery modes via smartphones include infographics (used to provide additional support for different topics), text messages (used to offer feedback messages), and social media discussions (ie, parents have the opportunity to communicate with members of the cohorts as well as experts in the WeChat group), which can be promising ways to provide parents with knowledge, goals, and advice to directly influence their children’s healthy lifestyles [30,54]. The primary outcome of this proposed study is preschoolers’ PA. PA has been considered dispensable and trivial because Chinese parents have subscribed to the concept that any activities, including PA, that compromise academic success and examination results should be eliminated [83]. This study may not only provide parents with the knowledge, skills, and confidence to create an environment for their children in which healthy behaviors (eg, PA, DB, and sleep) are developed and flourished, but also correct misleading concepts about PA. Parents’ PA, parenting style, and feeding practices are involved in secondary outcomes for several reasons. First, previous studies have indicated that preschoolers with parents who were regularly engaged in PA were more than 5 times more likely to be active than peers whose parents were not physically active, suggesting parents’ PA may be positively related to improved preschoolers’ PA [91]. Second, parenting style (ie, authoritative, authoritarian, permissive, and disengaged) is an important factor that potentially affects the lifestyle of young children. For example, an authoritative parental style was shown to be effective in improving healthy behaviors (ie, PA, diet, and less sedentary behaviors) [92]. Tyler et al [93] found that an authoritarian parental style was positively associated with preschoolers’ sleep problems. Third, parental feeding practices were associated with child eating patterns [94] because preschoolers’ food preferences and patterns have been shown to be influenced by the foods parents provide at a young age and food persistence in children’s meals [95].

This study has several strengths, including a randomized controlled trial design, calculation of sample size that ensures adequate statistical power, and objective and valid data collection methods for outcome measurements. Multiple lifestyle-related behaviors (ie, PA, diet, and sleep) are targeted in the intervention. The intervention delivery is grounded upon 4 behavioral modification steps included in SCT. A 3-month follow-up will determine whether the behavioral changes made during the intervention can be maintained. This study addresses many limitations summarized from previous studies, such as failure to either consider the imbalance of the intervention dose to bias the results or consider the sequence of the intervention contents to bias the results.

There are some limitations that need to be considered. The parent-child dyads that will be allocated to either the intervention or control group may be from the same school, and it is therefore possible that the intervention content may be contaminated. Thus, the outcome of this intervention may not be generalizable to preschoolers living in other cities in China. Owing to subjective measurements for the questionnaires, there is potential for parents to intentionally or unintentionally misreport; however, this is common in all free-living studies examining behavioral measures [96].

This study will make an important contribution to the literature on the application and verification of eHealth interventions (eg, social media) for cultivating healthy lifestyles (ie, PA, DB, and sleep) among Chinese preschoolers, where parents are the agents of change. By applying a theory-based (SCT) intervention with eHealth technology, a new perspective for future scientific health promotion will be obtained. As China has a large population, the implementation of a traditional face-to-face intervention to improve the unhealthy lifestyles of preschoolers would pose a heavy financial burden on society. According to the China Internet Network Information Center, as of December 2023, around 78% of Chinese people are connected to mobile devices with access to the internet (including those living in rural and remote regions), potentially enabling widespread access regardless of geographic location. This intervention has the potential to achieve a broad reach as it addresses many obstacles associated with the face-to-face delivery method. If the intervention is found to be efficacious, the prevalence of unhealthy lifestyles among preschoolers may be alleviated at a low cost, which not only has a positive influence on the health of individuals and the well-being of the family, but also reduces the financial pressure on society to treat diseases caused by poor lifestyle habits.

## Appendix

Multimedia Appendix 1 Intervention content of each module.

## Abbreviations

CPM: counts per minute
DB: dietary behavior
MET: metabolic equivalent task
PA: physical activity
SCT: social cognitive theory
SMART: Specific, Measurable, Achievable, Relevant, and Time-bound

## Data Availability

The data that support the findings of this study are available from the corresponding author upon reasonable request.
