# Supplementary material for: Effectiveness of a Parent-Based eHealth Intervention for Physical Activity, Dietary Behavior, and Sleep Among Preschoolers: Protocol for a Randomized Controlled Trial
Source: JMIR Res Protoc. 2024 Sep 12;13:e58344. doi: 10.2196/58344 (PMC11444123; doi:10.2196/58344)
Supplement: Multimedia Appendix 1 [file resprot_v13i1e58344_app1.docx]

**Multimedia Appendix 1.** Intervention content of each module.

| Week and module | Attention (personal factor); path: video and reading material | Retention (environmental factor); path: interaction and discussion in the WeChat group | Reproduction (behavioral factor); path: current barriers, suggestions, and goal setting | Motivation (behavioral factor); path: feedback and goal adjustment |
| --- | --- | --- | --- | --- |
| Week 1 (PA^a^): Developing basic motor skills and reaching PA goals | What is PA? What is the difference between PA and physical education? Why is it good for children? Current PA condition of preschoolers. How much PA should children get? How can children get moving more? Goal setting and tips. | Example: What is your child’s current PA patterns? How much time does your child spend on the TV, tablet, or smartphone? | Barriers: My child often remains sedentary at home and school. He or she does not like PA. Homework discourages my child to engage in PA. | Example: A consistent structured environment, where family members supported children in choosing an optimally challenging and realistic goal, breaking down goals into proximal (daily) and distal (weekly) PA targets, and providing praise and positive feedback on progress toward those goals. Time spent on screen time or sedentary behaviors can be reduced. Be physically active during the class recess or after school. |
| Week 2 (diet): Components of a healthy diet | Introduction to healthy eating. How much food consumed is good enough? Dietary structure regulations for preschool children. Goal setting and tips. | Example: How much food does the parent serve for the child? Does the parent read the ingredient label of the foods while buying? | Barriers: My child consumes less core food (eg, wheat and grain). My child consumes less protein and vitamins. | Example: Increasing the proportion of fruit and vegetable consumption in daily life. Adjusting serving size. Recipe modification. |
| Week 3 (sleep): Ensuring adequate sleep for preschool children | Why sleep is important for children’s development? Sleep characteristics of preschoolers. The drawbacks of sleep deprivation. Recommended sleep duration for preschool children. What can parents do? Goal setting and tips. | Example: What is your child’s sleeping pattern now? When does your child sleep every day? | Example: My child often refuses to sleep at night. | Example: Parents set a regular nightly bedtime for their young children. |
| Week 4 (PA): Reducing sedentary time and increasing PA | Introduction of sedentary behaviors. The dangers of sedentary behavior. The current situation of sedentary behavior among young children. Recommended sedentary time for preschoolers. Goal setting and tips. | Example: How much time do parents spend on PA with their child? | Barriers: My child likes to be sedentary. My child prefers to watch TV when finishing homework. | Example: Parents can replace their children’s sedentary time at home, especially electronic screen use, by establishing physical play. Parents can get up every 30 minutes to physically stretch with their children while he or she reads and draws. Replace sedentary time by standing whenever possible. Engagement in PA instead of sedentary behaviors. |
| Week 5 (Diet): Healthy eating and rational food eating | What kinds of food should preschoolers have? How much food should preschoolers have? When should preschoolers have their meals and snacks? How can parents provide healthy meals? Goal setting and tips. | Example: What snacks and drinks are consumed in your house? Does your child drink juice instead of eating fruit? | Barriers: My child buys snacks every time when going shopping. | Example: Parents can make a plan to buy the ingredients needed to make snacks and lead children in the process of buying in the supermarket to explain to the children the ingredients list of the food, which ingredients are healthy, and which ingredients are harmful to the body so that children can develop the ability to identify the nutrition of food. |
| Week 6 (sleep): Effective approaches to help preschool children fall asleep | What are the consequences of sleep deficits? What symptoms are there in children with sleep disorders? Sleep recommendation; improving sleep environment; goal setting; and tips. | Example: Are there any sleep problems in your child (eg, nightmares)? | Example: My child snores or dream talks sometimes. | Example: Parents can help their children sleep by telling them stories before they go to sleep and not offering any food before bedtime. |
| Week 7 (PA): Providing a wide variety of exercise options to increase PA | PA and intellectual development; variety of sports options | Example: Can your child perform motor tasks? | Barriers: My child has difficulty performing motor tasks. | Example: Parents should provide children with a variety of movement environments, so that they can fully experience the changes in the indoor and outdoor environments, as well as the differences between different movement interfaces, such as the ground, water, ice, and snow. |
| Week 8 (diet): Reducing intake of sugar-sweetened beverages | What are the sweetened beverages? Large amounts of vitamin C and many minerals (such as iron, potassium, and magnesium) are lost in the process of juicing fruits and vegetables. Moreover, juicing leads to a significant reduction in dietary fiber in the fruit itself. Goal setting and tips. | Example: Introduction of the golden rule: Parents decide what, when, and how the child eats; the child decides whether and how much they eat. | Barriers: Parents serve a lot of juice instead of providing fruits and vegetables directly. | Example: Limit consumption of foods and beverages containing large amounts of sugar. Parents should try to encourage their toddlers to eat fruits and vegetables directly to help them develop healthy eating habits. Parents also need to know not to buy processed drinks for young children and not to store them in the home. |
| Week 9 (sleep): Sleep environment | What is the optimal sleep environment for children? Why is the environment important to sleep? What can parents do to create a cozy sleep environment for their child? Goal setting and tips. | Example: Being in a dark room is very good for preschoolers to fall asleep? | Barriers: My child often gets tired in the daytime even though he or she has gotten enough sleep at night. | Example: Parents are advised to create a dark environment while their children are sleeping and not to allow their children to use electronic screens before bedtime, create a quiet environment for children to sleep, and read books or sing softly to their children. |
| Week 10 (PA): Parental modeling to increase PA | PA and mental health. What should parents do when it comes to increasing PA in young children? Parents should be actively involved in the physical activities of young children. | Example: Do you often do exercises with your children when you are free? | Barriers: My child does not like to play outdoor activities. | Example: Parents can take 20 minutes of their own free time to work with their children indoors or outdoors on light, moderate, or vigorous activities. |
| Week 11 (diet): Parenting feeding behaviors | Increase parental knowledge about the adverse effects of pressuring children to eat and offer food as a reward. Increase parental knowledge about alternative positive feeding practices to increase children’s interest and acceptance of healthy foods. Help parents identify child-related barriers regarding food refusal and how to overcome them using positive feeding practices. Foster parental self-regulation and self-efficacy to achieve changes in children’s food intake and parental feeding practices according to their needs. Goal setting and tips. | Are you often resorting to fast food for a quick bite to eat? | Barriers: Advertisements attract my child with their delicious and visually appealing food slogans. | Example: Are parents achieving goals? Weekly informative feedback (child’s food intake). Reinforcement and encouragement. Parents may not use food as a reward. Do not force the child to eat. Taking the child’s satiety into consideration. Role modeling. Provision of information on appropriate foods, food texture, tastes of food, and portion sizes. |
| Week 12 (sleep): The impact of parenting styles on young children’s lifestyles | Classification of parenting styles. Health consequences of sleep deprivation. The impact of different parenting styles on young children’s lifestyles. Goal setting and tips. | Example: How much does your child sleep every day? Does your child appear tired and sluggish during the daytime? | Barriers: My child is energetic and takes a long time to fall asleep. | Example: Being physically active during the day helps the child fall asleep more easily at night. Parents should establish a parenting style that centers on the healthy development of the child (ie, authoritative parenting style). |

^a^PA: physical activity.
